# Analysis of icatibant for the treatment of laryngeal hereditary angioedema attacks in the FAST-3 study

**DOI:** 10.1186/1710-1492-10-S2-A51

**Published:** 2014-12-18

**Authors:** William Yang, Jacques Hébert, Bruce Ritchie, Jovanna Baptista, Marc Riedl, William R Lumry

**Affiliations:** 1 Allergy & Asthma Research Centre, Ottawa, ON, Canada; 2 Centre de Recherche Appliquée en Allergie de Québec, Québec City, QC, Canada; 3 University of Alberta, Edmonton, AB, Canada; 4 Shire, Lexington, MA, USA; 5 University of California - San Diego, La Jolla, CA, USA; 6 AARA Research Center, Dallas, TX, USA

## Background

The efficacy and safety of icatibant for the treatment of edematous hereditary angioedema (HAE) attacks was established in three Phase III trials, including the For Angioedema Subcutaneous Treatment-3 study (FAST-3; NCT00912093). Here, data from the double-blind, controlled phase and open-label extension (OLE) of FAST-3 were analyzed post-hoc to specifically evaluate icatibant for the treatment of laryngeal attacks, which can cause potentially fatal airway obstruction.

## Methods

Controlled phase: adults with HAE type I/II were randomized to a single subcutaneous injection of icatibant 30 mg or placebo for their first mild-to-moderate laryngeal attack or moderate-to-very severe cutaneous/abdominal attack; severe laryngeal attacks were treated with open-label icatibant. OLE: attacks were treated with up to three icatibant injections, at ≥6-hour intervals. Three outcomes were analyzed for first icatibant-treated laryngeal attacks: 1) time to onset of symptom relief (earliest of three consecutive measurements for which there was a 50% reduction of patient–assessed 5-symptom composite 100 mm visual analog scale [VAS]); 2) time to almost complete symptom relief (earliest of three consecutive measurements for which all VAS symptom scores <10 mm); 3) patient- and investigator-assessed time to initial symptom improvement. Reinjection rates were also recorded.

## Results

For first icatibant-treated laryngeal attacks in the controlled phase or OLE (n=27), median (95% CI) time to onset of symptom relief was 2.0 (1.5-3.5) hours, median (95% CI) time to almost complete symptom relief was 6.0 (3.0–24.3) hours, and median (95% CI) patient- and investigator-assessed time to initial symptom improvement was 0.7 (0.4-0.9) and 0.8 (0.5-1.1) hours, respectively. In the OLE, 41/43 (95.3%) laryngeal attacks were treated with one icatibant injection, 2/43 (4.7%) were treated with two injections, and none required three injections.

## Conclusions

Icatibant provided symptom relief for mild-to-severe laryngeal HAE attacks in FAST-3. In the OLE, almost all laryngeal attacks were treated with one injection only.

